# Quality of life and associated factors among HIV positive patients after completion of treatment for Cryptococcal meningitis

**DOI:** 10.1371/journal.pntd.0008983

**Published:** 2021-03-03

**Authors:** Jonathan Kitonsa, Julius Kiwanuka, Zacchaeus Anywaine, Sheila Kansiime, Kenneth Katumba, Namirembe Aeron, Justin Beardsley, Freddie Kibengo, Alastair Gray, Pontiano Kaleebu, Jeremy Day

**Affiliations:** 1 Medical Research Council / Uganda Virus Research institute & London School of Hygiene and Tropical Medicine Uganda Research Unit, Entebbe, Uganda; 2 Department of Epidemiology and Biostatistics, School of Public Health, Makerere University Kampala, Uganda; 3 Oxford University Clinical Research Unit, Wellcome Trust Major Overseas Programme Vietnam, Ho Chi Minh City, Vietnam; 4 Marie Bashir Institute, Westmead Clinical School, University of Sydney, Sydney, Australia; 5 Health Economics Research Centre, Nuffield Department of Population Health, University of Oxford, Oxford, United Kingdom; 6 Nuffield Department of Clinical Medicine, University of Oxford, Old Road Campus, Headington, Oxford,United Kingdom; National Institute for Communicable Diseases, Johannesburg, South Africa, SOUTH AFRICA

## Abstract

**Background:**

Cryptococcal meningitis (CCM) remains one of the leading causes of mortality among HIV infected patients. Due to factors such as the severity of CCM pathology, the quality of life (QOL) of patients post-treatment is likely to be poor. Few studies have reported on QOL of CCM patients post treatment completion. We used data collected among patients in the CryptoDex trial (ISRCTN59144167) to determine QOL and associated factors at week 10 and six months from treatment initiation.

**Methodology:**

CryptoDex was a double-blind placebo-controlled trial of adjunctive dexamethasone in HIV infected adults with CCM, conducted between 2013 and 2015 in six countries in Asia and Africa. QOL was determined using the descriptive and Visual Analog Scales (VAS) of the EuroQol Five-Dimension-Three-Level (EQ-5D-3L) tool. We derived index scores, and described these and the VAS scores at 10 weeks and 6 months; and used linear regression to determine the relationship between various characteristics and VAS scores at both time points. VAS scores were interpreted as very good (81–100), good (51–80), normal (31–50) and bad/very bad (0–30).

**Results:**

Of 451 patients enrolled in the trial, 238 had QOL evaluations at week 10. At baseline, their mean age (SD) was 35.2(8.5) years. The mean index scores (SD) were 0.785(0.2) and 0.619(0.4) among African and Asian patients respectively at week 10, and 0.879(0.2) and 0.731(0.4) among African and Asian patients respectively at month six. The overall mean VAS score (SD) at 10 weeks was 57.2 (29.7), increasing significantly to 72(27.4) at month six (p<0.001). At week 10, higher VAS score was associated with greater weight (p = 0.007) and being African (p<0.001), while lower VAS score was associated with positive yeast culture at day 14 (p = 0.026). At month six, higher VAS score remained associated with African origin (p = 0.006) while lower VAS score was associated with positive yeast culture (p = 0.006). Lower VAS scores were associated with higher number of inpatient days at 10 weeks and 6 months (p = 0.003 and 0.002 respectively).

**Conclusion:**

QOL was good among patients that had completed therapy for CCM, but below perfect. Strategies to improve QOL among CCM survivors are required.

## Author summary

In spite of the remarkable reduction in Cryptococcal meningitis (CCM) related morbidity and mortality with increased use of antiretroviral therapy, incidence remains unacceptably high especially in sub-Saharan Africa and Asia where more than 90% of the cases and deaths occur.

Due to factors such as the severity of CCM pathology, the quality of life (QOL) of patients post-treatment is also likely to be poor. Few studies have reported on QOL of CCM patients post treatment completion. We used data collected among patients in the CryptoDex trial (ISRCTN59144167) to determine self-perceived QOL and associated factors among 238 survivors at week 10 and 203 survivors at six months from treatment initiation. We determined QOL using the descriptive and Visual Analog Scales (VAS) of the EuroQol Five-Dimension-Three-Level (EQ-5D-3L) tool. We found that while self-perceived QOL was only relatively good among this cohort of patients who had survived through treatment for CCM, it continued to improve over the 6 months following diagnosis. Low weight at diagnosis, prolonged hospital admission, positive yeast culture at day 14/treatment completion, and being Asian were associated with lower QOL. QOL is an important outcome that should be considered among HIV infected patients treated for serious infections such as CCM.

## Introduction

Cryptococcal meningitis (CCM) is one of the leading causes of mortality among HIV infected patients, especially in sub-Saharan Africa and Asia. Mortality from CCM remains high, in spite of a significant reduction in the incidence of opportunistic infections (OIs), resulting from improvement in HIV management including early initiation of antiretroviral therapy (ART) [1]. Between 2009 and 2014, the estimated global annual incidence and mortality due to CCM reduced from 975,900 to 223,100 and 624,700 to 181,100 respectively [2,3]. While incidence, prevalence, and mortality indices have been used widely to demonstrate the burden of CCM amongst HIV infected patients, its effects on other important measures of health such as quality of life (QOL) have not been adequately evaluated. Due to the advanced stage of AIDS at which CCM occurs, severity of disease pathology, toxicity of antifungal medications and disease sequelae, the QOL post-treatment is likely to be poor. We previously showed that patients with low QOL at treatment completion are more likely to die two years later than those with higher QOL[4] (this analysis included some participants reported on in this paper, particularly those from Uganda). Previous research has demonstrated that QOL measurements amongst HIV infected patients should be done routinely [5–8], since improved QOL is one of the ultimate goals of HIV management. We used data collected from patients enrolled in the CryptoDex trial (PMID: 25391338, ISRCTN59144167) [9,10] to determine QOL at 10 weeks and six months since treatment initiation and the factors associated with high or low QOL at 10 weeks and six months. We hypothesised that QOL would increase between 10 weeks since treatment initiation and six months among patients enrolled in the CryptoDex trial.

## Methodology

### Ethics statement

Ethical approval was obtained from: OxTREC, Oxford, UK 25–12; Ministry of Health, Vietnam; Hospital for Tropical Diseases, Ho Chi Minh City, Vietnam; Ministry of Health Laos 039/NECHR; Faculty of Medicine, Universitas Indonesia, Indonesia 623/H2.F1/ETIK/2012; Mahidol University, Thailand MUTM 2012-051-01; Institute for the Development of Human Research Protections, Thailand 04CN; Uganda Council for Science and Technology HS1264; National Drug Authority, Uganda 020/ESR/NDA/DID-01/2013; Ministry of Health, Malawi #1077; Pharmacy, Medicines and Poisons Board, Malawi PMPB/CTRC/111/3105201354; University of Toronto, Canada 28199.

Written consent was obtained from all patients or their representatives.

### Study design and setting

The CryptoDex trial was a double-blind placebo-controlled phase III trial of adjunctive dexamethasone in HIV infected adults with cryptococcal meningitis. The study was conducted between 2013 and 2015 at thirteen sites in six countries in Asia and Africa (Vietnam, Thailand, Indonesia, Laos, Uganda, and Malawi).

### Subjects enrolled in the CryptoDex study and their management

Patients recruited in the CryptoDex study were 18 years and above, had HIV infection, a clinical syndrome consistent with CCM, and microbiological confirmation of disease, as indicated by one or more of the following test results: 1) positive India ink staining of cerebrospinal fluid (CSF); 2) culture of *Cryptococcus* species from CSF or blood; or 3) cryptococcal antigen detected in CSF on cryptococcal antigen lateral flow assay (IMMY). Exclusion criteria included pregnancy, renal failure, gastrointestinal bleeding, having been treated with more than 7 days of anticryptococcal antifungal therapy, and being on or requiring glucocorticoid therapy for coexisting conditions. Informed consent was obtained from all patients or a next of kin where a patient was not in position to consent. Eligible patients were randomised in a 1:1 ratio, stratified by site, to receive either dexamethasone adjunctive therapy or placebo in a tapered dose until 42 days post-randomisation. Details of other study procedures and investigations can be found in the CryptoDex study protocol [11].

The treatment regimen included an induction phase with intravenous Amphotericin B (1 mg/kg/day) and oral Fluconazole 800mg daily for 14 days, followed by a continuation phase of 800mg daily fluconazole for eight weeks, and then a maintenance phase with oral fluconazole 200mg daily. This regimen was consistent with the WHO treatment recommendation for settings where Flucytosine was not available at the time the trial was conducted [12].

Patients were followed up in the study until six months after enrolment, with all study visits conducted at the study clinics or in hospitals for patients under admission. At six months, participants were exited and referred back to their HIV care clinics for continuation of HIV care.

### Subjects included in this analysis

This QOL analysis includes patients who survived up to 10 weeks post-treatment initiation when the first QOL assessment was performed. A second QOL assessment was done at month six and therefore patients who had not died between 10 weeks and six months and were accessible i.e., able to come to the clinics, are included in the six months analysis.

### Variables and their measurement in the cryptodex study

Quality of life was measured using the EQ-5D-3L tool [13]. This tool gives a subjective measure for the QOL, which is in line with the definition of QOL by WHO. The WHO defines QOL as an individuals’ perception of their position in the context of culture and value systems in which they live and in relation to their goals, expectations, standards, and concerns [14]. The tool is a widely used measure of health status consisting of two parts. The first part (the descriptive system) assesses health in five dimensions (mobility, self-care, usual activities, pain/discomfort, and anxiety/depression). Each dimension has three levels of response (no problems, some problems, extreme problems/not able to). This part of the EQ-5D-3L questionnaire provides a descriptive profile that can be used to generate a health state profile. For example, a patient in health state 11233 would have no problems in mobility and self-care, some problems with usual activities, severe/extreme pain/discomfort, and extreme anxiety or depression. Each health state is assigned a summary index score based on societal preference weights for the health state. Heath state index scores generally range from less than 0 (where 0 is a health state equivalent to death; negative values are valued as worse than death) to 1 (perfect health). Health state preferences can differ between countries. Where a value set is not available for a country/region, the assessor can opt to select a value set for a country/region considered to most closely approximate the country of interest [13]. We used the Thailand value set for sites in Asia (Vietnam, Thailand, Indonesia and Laos) [15] and the Zimbabwe value set for sites in Africa (Uganda and Malawi) [16]. The second part of the EQ-5D-3L questionnaire consists of a visual analog scale (VAS) on which the patient rates his/her self-perceived health from 0 (the worst imaginable health) to 100 (the best imaginable health/perfect health).

The tool was translated into the local languages and administered by a study doctor who read the questions to the patient. Other details concerning its administration can be found in the study protocol [11].

### Data analysis

All data from the CryptoDex study was derived from the secure OUCRU proprietary CLiRES trial database. Data were extracted into a Microsoft Access database for export to STATA (College Station, TX, version 15.0). Categorical variables were summarised by frequencies and percentages, while continuous variables were summarised using means (standard deviations) and/or medians (interquartile ranges).

A health profile was generated by continent and time points (i.e., week 10 and month six), from which we derived a health state index score using the selected value sets for each region. We then summarised the index scores by means, standard deviations (SD), and minimum and maximum scores by continent at the different time points. VAS scores were interpreted as very good (81–100), good (51–80), normal (31–50) and bad/very bad (0–30) [17]. T-tests were used to compare VAS scores between continents and to test for significant changes in VAS scores and index scores at the different time points among different variables. We also computed and presented percentages of categories under each of the five dimensions of the EQ-5D-3L questionnaire.

We used linear regression to assess the impact of a number of variables on QOL measured using the VAS scores at week 10 and month six including: demographic characteristics (age, sex, and education measured as number of years in school); Glasgow Coma Score (GCS) continent, i.e. Asia or Africa; ART status, i.e. on or not on ART; time on ART; most recent CD4 count at enrolment; presence of convulsions; intervention arm, i.e., dexamethasone or placebo; Number of inpatient days by week 10 and month six (i.e., including days of readmission after the first hospitalisation), baseline CSF fungal burden, yeast culture at 14 days, and Number of adverse events (neurological events, new AIDs defining illnesses, other adverse events, and inflammatory immune reconstitution syndrome events).

This analysis was not repeated using index scores as two value sets (Thailand and Zimbabwe) were used, and sample sizes were too small to conduct separate analyses. Variables that had a p-value of 0.2 and below at univariate analysis were included in a multivariable linear regression model. Variables were retained in the multivariable model if their inclusion did not make the model significantly worse at LRT p-value 0.05.

## Results

### Baseline characteristics

**Fig 1:**
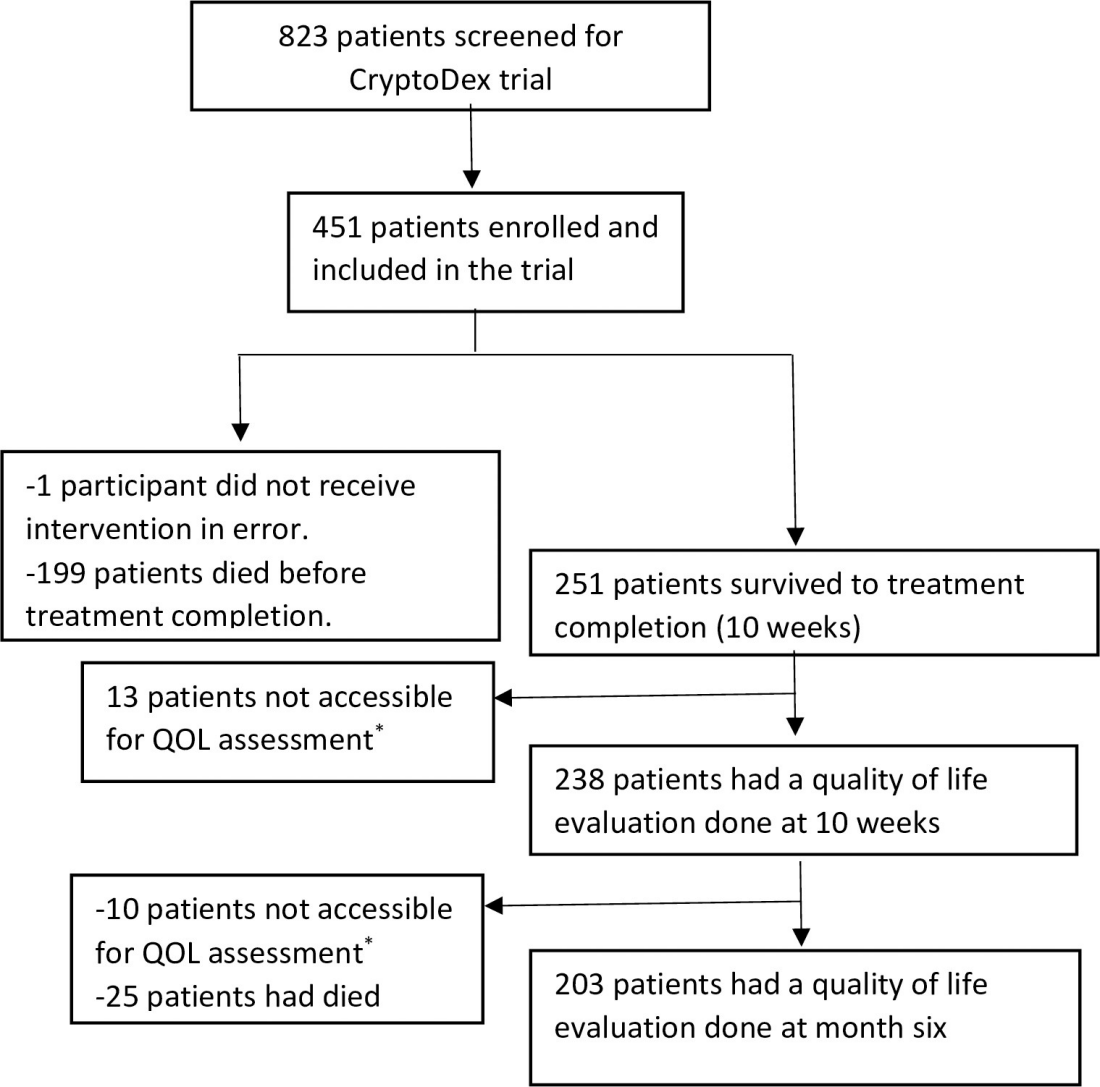
Flow diagram showing participants enrolled in CryptoDex trial who had quality of life evaluations done. * Patients were not able to come to clinics to have QOL assessment.

Fig 1 is a flow diagram showing the patients included in this analysis. Out of 823 patients screened at the 13 sites globally, 451 were found eligible and randomized. One patient was subsequently excluded because he never received the assigned study drug due to an administrative error. Out of the remaining 450 patients, 251 (55.8%) survived to week 10 post-treatment initiation, and 238 (94.8%) completed a QOL assessment and were included in our analysis. The other 13 (5.2%) patients were not accessible for the quality of life assessment since they did not come to the clinics. A comparison of baseline characteristics among the 238 participants included in the analysis and the 213 not included is shown in S1 Table. At baseline, the mean age (SD) of the 238 patients was 35.2 (8.5) years, and 101 (42.4%) were on ART. The median CD4 count (IQR) was 30 (13, 71) cells/ml. Thirty (12.6%) patients reported a history of convulsions. Of the 238 patients, 113 (47.5%) had been randomised to receive dexamethasone adjunctive therapy. The baseline characteristics are summarised in Table 1.

**Table 1:** Baseline characteristics of the patients that had QOL evaluation at 10 weeks.

		Overall	Africa	Asia
Variable	Level	Frequency (%)	Frequency (%)	Frequency (%)
Gender (n = 238)	**Male**	152(63.9)	71(57.3)	81(71.1)
	**Female**	86(36.1)	53(42.7)	33(29.0)
Mean age (SD), (n = 238)		35.2(8.5)	34.9(8.7)	35.7(8.3)
Median CD4 (IQR), (n = 126)*		30(13, 71)	40(15, 104)	25(9, 51)
Years in school (n = 215)**	**≤7**	107(49.8)	63(57.3)	44(41.9)
	**>7**	108(50.2)	47(42.7)	61(58.1)
Glasgow coma Score (n = 238)	**15**	210(88.2)	107(86.3)	103(90.4)
	**<15**	28(11.8)	17(13.7)	11(9.6)
Median Period known to have HIV in years (179) (IQR)		0.5(0.1, 2.9)	0.5(0.2, 2)	0.2(0.1, 1.9)
On ARVs (n = 238)	**No**	137(57.6)	53 (42.7)	84(73.7)
	**Yes**	101(42.4)	71(57.3)	30(26.3)
Confusion (n = 238)	**No**	180(75.6)	83(66.9)	97(85.1)
	**Yes**	58(24.4)	41(33.1)	17(14.9)
Mean Weight (SD) (n = 238)		52.1(10.1)	52.7(10.8)	51.5(9.3)
Convulsion (n = 238)	**No**	208(87.4)	117(94.3)	91(79.8)
	**Yes**	30(12.6)	7(5.7)	23(20.2)
Intervention (n = 238)	**Dexamethasone**	113(47.5)	55(44.4)	58(50.9)
	**Placebo**	125(52.5)	69(55.6)	56(49.1)
CSF Culture, CFU/ml (n = 227)***	**<1000cfu/ml**	78(34.4)	47(39.8)	31(28.4)
	**≥1000cfu/ml**	149(65.6)	71(60.2)	78(71.6)

*Pre-enrolment CD4s considered. CD4s were not repeated in the study. Some patients had no record of CD4 counts.

**Responses could not be got for some participants because of altered consciousness.

***Lumbar punctures were not successful in a few patients.

### Adverse events

Four hundred and thirty-four adverse events were captured among these participants, including 67 new neurological events, 93 new AIDs defining illnesses, 365 other adverse events, and 9 immune reconstitution syndrome events.

### Quality of life assessment

#### Descriptive system and index scores

Health profile results for patients at both time points are summarised in Table 2. There was no difference in the proportion of patients from Africa or Asia rating themselves as having perfect health (i.e., profile 11111) at 10 weeks (Africa: 40 of 124 (32.3%); Asia: 37 of 114 (32.5%)). At this time point, 4 Asian patients (3.5%) rated themselves as having the worst health state possible i.e. 33333 compared with 1 African patient (0.8%, p = 0.2). At month six, reported quality of life had increased in both African and Asian centres, with 58 of 104 (55.8%) Africans and 48 of 99 (48.5%) Asians rating themselves as having perfect health (p = 0.2).

**Table 2:** Health profile scores for patients at week 10 and month six.

CONTINENT	Level	Mobility		Self-care		Usual activities		Pain/discomfort		Anxiety/ depression	
		Week 10 n (%)	Month six n (%)	Week 10 n (%)	Month six n (%)	Week 10 n (%)	Month six n (%)	Week 10 n (%)	Month six n (%)	Week 10 n (%)	Month six n (%)
AFRICA	**1(no problems)**	**79(63.7)**	**84(80.8)**	**102(82.3)**	**99(95.2)**	**59(51.8)**	**72(69.2)**	**72(58.1)**	**75(72.1)**	**78(63.4)**	**79 (76)**
	**2(some problems)**	**39(31.5)**	**20(19.2)**	**14(11.3)**	**4(3.8)**	**35(30.7)**	**25(24.1)**	**47(37.9)**	**28(26.9)**	**37(30.1)**	**21(21.1)**
	**3(extreme problems)**	**6(4.8)**	**0**	**8(6.4)**	**1(1)**	**20(17.5)**	**7(6.7)**	**5(4)**	**1(1)**	**8(6.5)**	**3(2.9)**
ASIA	**1(no problems)**	**71(62.3)**	**72(72.7)**	**83(72.8)**	**85(85.9)**	**59(51.8)**	**68(68.7)**	**60(53.1)**	**61(61.6)**	**76(68.5)**	**69(70.4)**
	**2(some problems)**	**28(24.5)**	**22(22.2)**	**16(14)**	**8(8.1)**	**35(30.7)**	**20(20.2)**	**42(37.2)**	**33(33.3)**	**28(25.2)**	**22(22.5)**
	**3(extreme problems)**	**15(13.2)**	**5(5.1)**	**1 (13.2)**	**6(6)**	**20(17.5)**	**11(11.1)**	**11(9.7)**	**5(5.1)**	**7(6.3)**	**7(7.1)**

**Abbreviations:** n, Number; %, percentage.

At week 10, the mean index scores (SD, range) were 0.785 (0.2, -0.145 to 1) and 0.619 (0.4, -0.452 to 1) for African and Asian patients respectively, increasing significantly at month six, to 0.879 (0.2, 0.341 to 1), p = 0.002 and 0.731 (0.4, -0.452 to 1), p = 0.052 for African and Asian patients respectively.

### EQ VAS scale results

The overall mean VAS score (SD) at 10 weeks was 57.2 (29.7), increasing significantly to 72 (27.4) at month six (p<0.001), both mean scores falling within category 51–80, i.e. “Good QOL”. The mean score (SD) at 10 weeks was significantly higher among African patients than Asian patients i.e., 65.7 (21.5) vs 47.5 (34.5), p<0.001. Similarly, the mean score (SD) at six months was significantly higher among African patients compared to Asian patients i.e., 79 (17.7) vs 64.2 (33.4), p<0.001.

The mean (SD) VAS scores at 10 weeks among those that received dexamethasone and placebo were not statistically significantly different, i.e., 54.5 (30.4) and 59.4 (29), p = 0.22 respectively.

Similarly, there was no statistically significant difference in mean VAS score at month 6 among those that received dexamethasone and placebo i.e., 69.1 (28.7) and 74.2 (26.1), p = 0.193, respectively.

**Fig 2:**
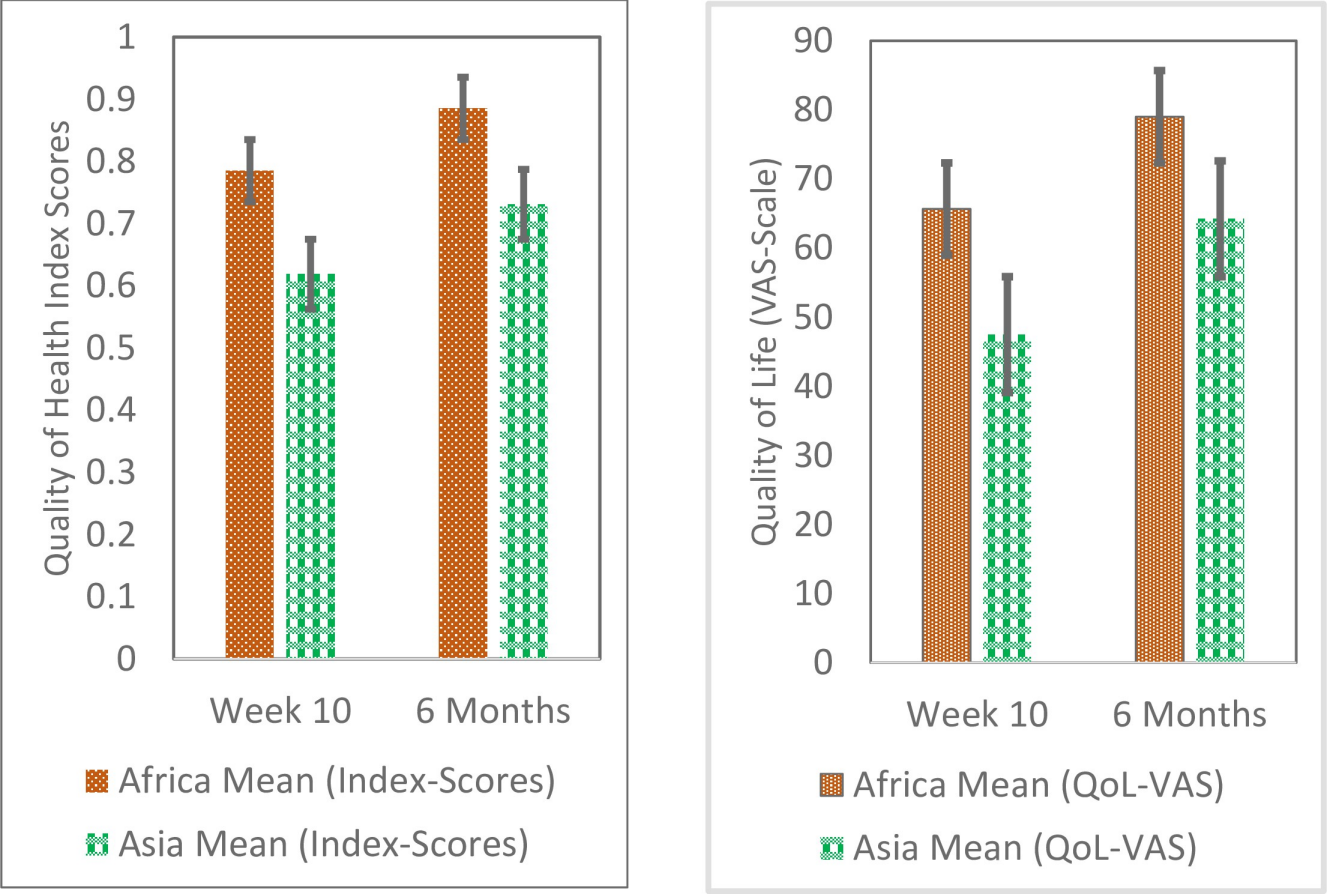
Mean VAS scores and Index scores at week 10 and 6 months after treatment initiation. Abbreviations: VAS, Visual analog scale; QoL, Quality of life.

Mean VAS and Index scores at week 10 and month six are represented in Fig 2. There was a statistically significant increase in mean VAS between week 10 and month six on all variables including gender (16.0, p<0.001 among males; 13.5, p<0.001 among females), GCS at baseline (14.4, p<0.001 among “GCS = 15”; 20.6, p<0.001 among “GCS<15”), confusion at baseline (12.9, p<0.001 for “no confusion”; 21.6, p<0.001 for “confusion present”), Convulsions at baseline (14, p<0.001 among “convulsions present”; 21, p<0.001 among “convulsions absent”), intervention (13.9, p<0.001 for placebo; 16.6, p<0.001 for dexamethasone), CSF culture at baseline (15.2, p<0.001 for “<1000CFU/ml”; 16, p<0.001 among “≥1000CFU/ml”), and CSF culture at day 14(14.3, p<0.001 among “0 CFU/ml”; 16.3, p<0.001 for “>0CFU/ml”), and continent (13, p<0.001 for Africa; 16.9, p<0.001 for Asia).

### Factors associated with quality of life

Results from the univariate and multivariate analysis are summarised in Table 3. At week 10, the baseline factors associated with higher VAS score at multivariate analysis were: greater weight (regression coefficient 0.6, 95% CI: 0.2 to 1.1), p = 007; and being from Africa (regression coefficient 19.4, 95% CI: 9.8 to 29), p<0.001. Higher inpatient days by week 10 was associated with poorer VAS score (regression coefficient -0.6, 95% CI -1.1 to -0.2), p = 0.003. Similarly, positive yeast culture at day 14 was associated with poorer VAS score (regression coefficient -11.7, 95% CI -21.9 to -1.4), p = 0.026. There was no relationship between VAS score at week 10 and presence of confusion, convulsions, CSF pressure, and fungal burden at baseline.

**Table 3:** Quality of Life and associated factors at 10 weeks and 6 months.

		WEEK 10				MONTH SIX			
		Univariate linear regression		Multivariate linear regression		Univariate linear regression		Multivariate linear regression	
Variable	Categories	Reg. Coef. (95% CI)	P Value	Reg. Coef. (95% CI)	P Value*	Reg. Coef. (95% CI)	P Value	Reg. Coef. (95% CI)	P Value*
**Gender**	**Male**								
	**Female**	6(-2.1 to 14.1)	0.145	1.0(-8.7 to 10.8)	0.834	2.8(-5.2 to 10.8)	0.497		
**Age**	***Cont*.**	-0.1(-0.5 to 0.4)	0.852			-0.4(-0.9 to 0.2)	0.173	-0.2(-0.7 to 0.3)	0.558
**Years in School**	**≤7**								
	***>7***	-0.7(-9 to 7.6)	0.862			-2.2(-10.4 to 5.9)	0.588		
**Glasgow coma Score**	**<15**								
	**15**	7.2(-4.6 to 19.0)	0.230	1.8(-11.7 to 15.3)	0.795	3.7(-8.3 to 15.7)	0.547		
**ART at Baseline**	**Yes**								
	**No**	-6.2(-14.0 to 1.6)	0.121	4(-5 to 12.9)	0.383	-7.7(-15.4 to 0.03)	0.051	2.9(-6.9 to 12.8)	0.557
**Confusion**	**Yes**								
	**No**	7.2(-1.7 to 16.1)	0.114	5.2(-6 to 16.3)	0.362	-1.2(-10.1 to 7.6)	0.789		
**Weight**	***Cont*.**	0.5(0.2 to 0.9)	0.005	0.6(0.2 to 1.1)	0.007	0.2(-0.2 to 0.5)	0.397		
**Convulsion**	**Yes**								
	**No**	11.9(0.3 to 23.4)	0.044	-2(-14.4 to 10.3)	0.745	5.5(-6.4 to 17.2)	0.366		
**Inpatient days by week 10**	***Cont*.**	-0.7(-1.1 to -0.4)	<0.001	-0.6(-1.1 to -0.2)	0.003	N/A	N/A		
**Inpatient days by month six**	***Cont*.**	N/A	N/A	N/A	N/A	-0.5(-0.7 to -0.2)	<0.001	-0.7(-1.2 to -0.3)	0.002
**Intervention**	**Dexa.**								
	**Placebo**	4.9(-2.9 to 12.6)	0.220	0.6(-7.9 to 9.1)	0.886	5.1(-2.6 to 12.8)	0.193	2.7(6.9 to 12.2)	0.581
**Continent**	**Asia**								
	**Africa**	18.2(10.8 to 25.6)	<0.001	19.4(9.8 to 29)	<0.001	14.8(7.4 to 22.2)	<0.001	15.6(4.5 to 26.7)	0.006
**CSF Culture, CFU/ml**	**<1000**								
	**≥1000**	-6.5(-14.7 to 1.6)	0.116	0.9(-7.1 to 8.9)	0.821	-4.9(-12.9 to 3.1)	0.231	5.1(-5.4 to 15.6)	0.338
**CSF culture day 14, CFU/ml**	**0**								
	**>0**	-17.7(-26.2 to -9.1)	<0.001	-11.7(-21.9 to -1.4)	0.026	-15.9(-24.3 to -7.5)	<0.001	-15.7(-26.8 to -4.7)	0.006
**Adverse events**	**None**								
	**1–3**	-1.0(-10.5 to 8.5)	0.841	7.1(-3.2 to 17.5)	0.178	-3.1(-12.3 to 6.0)	0.498	10.1(1.2 to 21.3)	0.078
	**4+**	-10.2(-22.0 to 1.6)	0.09	11.9(-2.9 to 26.6)	0.116	-10(-22.2 to 2.2)	0.107	17.6(-2.1 to 37.3)	0.079

**Abbreviations:** ART, Antiretroviral therapy; CI, Confidence interval; Reg, Regression; Coef, Coefficient; Cont. Continuous variable; N/A, not applicable; Dexa, Dexamethasone; CFU/ml, Colony forming units per millilitre.

* Only variables with a P Value <0.2 in the univariate analysis were used in the multivariable linear regression model.

At month six, African origin was associated with higher VAS score at multivariate analysis (regression coefficient 15.6, 95% CI 4.5 to 26.7), p = 0.006, while positive yeast culture at day 14 was associated with poorer VAS score (regression coefficient -15.7, 95% CI -26.8 to -4.7), p = 0.006. Again, higher inpatient days was associated with a reduced VAS score at 6 months (regression coefficient -0.7, 95% CI -1.2 to 0.3, p = 0.002. There was no relationship between VAS score at month six and presence of confusion, convulsions, CSF pressure, and fungal burden at baseline.

## Discussion

HIV-associated CCM is an extremely debilitating disease associated with high mortality. Affected patients present with advanced HIV disease and considerable immunosuppression. While the disease itself is associated with significant risks of sequelae including neurological deficits, the treatment as well, of which amphotericin is the backbone, is also associated with significant toxicities [18,19]. All these factors have the potential to reduce the QOL among patients who survive up to treatment completion.

We were pleased to see that many patients reported relatively good self-perceived QOL following completion of treatment for CCM, and that this tended to improve thereafter through the 6 months following diagnosis. However, given the nature of the disease and its treatment, it is perhaps not surprising that very few patients reported ‘very good’ or ‘perfect’ health. We did not assess QOL at study entry in the CryptoDex trial and thus could not measure improvement directly attributable to antifungal treatment. However, we found that patients reported significant improvements in QOL between 10 weeks and six months after diagnosis, measured both with the descriptive and VAS scales. These continuing improvements in QOL are likely due to multiple factors. These may include gradual improvement due to neurological recovery, better engagement with health services, and the use of antiretroviral drugs. A previous study identified that patients with stage 3 and 4 HIV disease had significant improvements in QOL after ART initiation [5].

It has been shown previously that different antifungal combinations can be associated with long term (6 month) differences in survival [20]. Therefore, while some of the improvement in QOL seen in our patients, who all received the same antifungal treatment, may be due to the factors we described above, developing more effective induction and consolidation therapy for CCM is likely to yield further improvements in QOL.

We consider QOL an important health parameter to measure since better scores have been shown to correlate with long term survival [4,21]. In our study, we used the EQ-5D-3L tool. This has the advantages of simplicity and ease of administration including amongst very sick patients, as well as ease of scoring and interpretation. While it has not been used previously in many studies of CCM patients, our work shows that it can identify changes in QOL over time following treatment of CCM. As such, it has the potential to identify patients who may benefit from increased follow-up during the recovery process–further studies are needed. Identifying such patients could help with distribution of scarce resources and ensure the best possible outcomes for all patients.

We found that prolonged admission by week 10 and month 6 were associated with lower QOL measured using the VAS at week 10 and month six. This is not surprising—patients admitted for longer periods were likely more ill at baseline. A similar finding was reported in a study done by Nafteux and colleagues in Belgium [22].

We were surprised to see that patients from African countries in the CryptoDex trial rated themselves to have better QOL at week 10 and six months than did Asian patients. This effect was seen with both the descriptive scale and VAS. This effect remained apparent in the adjusted model. We suggest that this is probably a societal perceptual variation rather than an indication of impact of disease. It may also be argued that the resilience developed by patients in the African setting may influence their opinion about ill health. They may not eventually perceive as equally bad ill health situations suffered as their Asian counterparts.

We found that higher weight was associated with higher VAS at week 10. The most likely explanation here is that low weight is correlated with more severe disease [23].

We also found that patients who had positive yeast culture at day 14, which also corresponded to the day of completion of Amphotericin therapy, had poorer quality of life at week 10 and month six. Research had previously shown that positive CSF culture at day 14 of Amphotericin was associated with poorer longer term outcomes including mortality and relapse [24–26]. However, Rolfes and colleagues demonstrated that these poor outcomes were prevented if 800mg of Fluconazole was used in the consolidation phase of treatment [27]. Our finding that positive CSF culture is associated with poor QoL even when Fluconazole was used at this dose deserves serious consideration.

Unlike a previous study from Uganda amongst HIV patients that found that male gender was associated with better quality of life, we did not identify a difference between males and females in terms of QOL following treatment for CCM [28].

Our analysis has some limitations. First, we used a subjective measure of QOL, which may not be an absolute indication of the QOL of an individual. Naturally, optimistic people may rate their health status higher than pessimistic ones. However, the WHO recommends that QOL tools should measure from the subjective viewpoint since the patient’s experience is more relevant than the assessment of an objective observer [29]. Second, we used health index scores derived from models of countries that we assumed were similar to our own, since models do not exist for all the countries in our study. Use of validated index scores from the actual countries that participated in CryptoDex could conceivably result in different outcomes. However, we think this is unlikely because we measured QOL at two time points and found improvement over time. QOL was not assessed at baseline in the CryptoDex trial. This would have been very informative since it would have enabled determination of the impact of treatment on QOL as well as a comparison of differences in QOL for those that got dexamethasone and those that did not. It would be interesting to determine the effect of some other factors that were not measured in this study such as one’s perception of their HIV disease and the presence or lack of social support.

The current doses of amphotericin, which clearly have the greatest antifungal effect, may in fact be resulting in patient harm because of associated toxicities [30]. These toxicities may contribute to lowering the quality of life of patients. QOL measurements among survivors could help in parsing such data, while we wait for more effective and tolerable novel treatments to be developed. Patients with attributes associated with poor quality of life identified here such as low weight and positive yeast culture at day 14 need special consideration. Useful interventions may include nutritional support and using culture results to inform discontinuation of therapy. Culture and sensitivity may also rule out possibility of resistance to drugs. Simple interventions such as counselling may help patients deal with issues such as anxiety. This may be supplemented by adherence counselling since CCM among patients on long-term ART is often a marker of treatment failure, often due to poor adherence. Going forward, we also recommend that QOL assessment are incorporated in the evaluation of patients with cryptococcal meningitis. The EuroQol Five-Dimension-Three-Level (EQ-5D-3L) tool used in this study and other tools such as the WHO’s WHOQOL-100 [29] may be appropriately used to generate useful data on quality of life. Qualitative QOL assessments may also provide useful information that could inform interventions.

## Conclusion

While self-perceived QOL was only relatively good among this cohort of patients who had survived through treatment for CCM, it continued to improve over the 6 months following diagnosis. Low weight at diagnosis, prolonged hospital admission, being Asian, and positive yeast culture at day 14 were associated with lower self-perceived QOL. QOL is an important outcome that should be considered among HIV infected patients treated for serious infections such as CCM.

## Supporting information

S1 Table Is showing comparison of baseline characteristics of the patients that had QoL evaluation at 10 weeks (n = 238) and those that did not (n = 213). (DOCX)
