# Supplementary material for: Quality of life and associated factors among HIV positive patients after completion of treatment for Cryptococcal meningitis
Source: PLoS Negl Trop Dis. 2021 Mar 3;15(3):e0008983. doi: 10.1371/journal.pntd.0008983 (PMC7959361; doi:10.1371/journal.pntd.0008983)
Supplement: S1 Table — (DOCX) [file pntd.0008983.s001.docx]

**S1_Table: Table showing comparison of baseline characteristics of the patients that had QoL evaluation at 10 weeks (n=238) and those that did not (n=213)**

| Characteristic | Group with QoL assessment at week 10 (N=238) | Group without QoL assessed at week 10 (i.e., Dead by week 10 or unable to come to clinic) (N=213) | P - value |
| --- | --- | --- | --- |
| Gender |  |  |  |
| Males (%) | 152 (63.87) | 128 ( 60.09) | 0.824 |
| Females (%) | 86 (36.13) | 85 (39.91) |  |
| Mean age (SD) | 35.2 (8.5) | 37.7 (8.1) | 0.01 |
| Median CD4 (IQR) | 30 (13,71) | 25 (9,62) | 0.233 |
| Glasgow coma score |  |  |  |
| 15 | 210 (88.2) | 154 (72.6) | <0.001 |
| <15 | 28 (11.8) | 58 (27.4) |  |
| Period known to have HIV (IQR) | 0.5(0.1,2.9) | 1.2(0.2,4) | 0.067 |
| On ARVs |  |  |  |
| No | 137 (57.6) | 132 (62) | 0.341 |
| Yes | 101 (42.4) | 81 (38) |  |
| Confusion |  |  |  |
| Yes | 58 (24.4) | 78 (37) |  |
| No | 180 (75.6) | 133 (63) |  |
| Mean weight (SD) | 52 (10) | 49 (9) | <0.001 |
| Convulsions |  |  |  |
| No | 208 (87.4) | 163 (77.3) | 0.005 |
| Yes | 30 (12.6) | 48 (22.7) |  |
| Intervention |  |  |  |
| Dexamethasone | 113 (47.5) | 111 (52.1) | 0.326 |
| Placebo | 125 (52.5) | 102 (47.9) |  |

**Abbreviations:** n, Number; ARVs, Anti retro viral therapy; SD, Standard deviation; HIV, Human immunodeficiency virus; %, percentage.
